# Whitening Efficiency of Papain and Bromelain Gels in Removing Dental Stains

**DOI:** 10.3390/gels11020100

**Published:** 2025-02-01

**Authors:** Stanca Cuc, Codruta Sarosi, Ioan Petean, Amalia Moldovan, Cecilia Bacali, Sorin Claudiu Man

**Affiliations:** 1 Department of Polymer Composites, Institute of Chemistry Raluca Ripan, Babeș-Bolyai University, 30 Fantanele Street, 400294 Cluj-Napoca, Romania; stanca.boboia@ubbcluj.ro; 2 Faculty of Chemistry and Chemical Engineering, Babeș-Bolyai University, 11 Arany Janos Street, 400028 Cluj-Napoca, Romania; petean.ioan@gmail.com; 3 Physics and Chemistry Department, Technical University of Cluj-Napoca, 28 Memorandumului Street, 400114 Cluj-Napoca, Romania; 4 Department of Prosthodontics and Dental Materials, Faculty of Dental Medicine, Iuliu Hatieganu University of Medicine and Pharmacy, 32 Clinicilor Street, 400006 Cluj-Napoca, Romania; cecilia.bacali@umfcluj.ro; 5 Mother & Child Department, Pediatrics III, Iuliu Hatieganu University of Medicine and Pharmacy, 2-4 Campeni Street, 400217 Cluj-Napoca, Romania; claudiu.man@umfcluj.ro

**Keywords:** tooth bleaching, enzyme, papain, bromelain, enamel surface morphology, bleaching gels

## Abstract

This study aimed to evaluate the micro-nanostructure and color changes of dental enamel after treatment with new gel formulations containing papain or bromelain. Eighty caries-free, extracted human teeth were randomly divided into two groups (n = 40) and stained by immersion in either coffee or Tedi juice for 4 h daily over five consecutive days. After staining, the samples were washed and stored in artificial saliva at 37 °C. The stained samples were then treated with four different whitening gels (GC, G1, G2, and Opalescence 15%) for 4 h daily. Following each treatment, the samples were rinsed and stored in artificial saliva. Color changes were measured using a digital spectrophotometer to assess CIEL*a*b* ΔE* and the Whiteness index (WI). The enamel micro-nanostructure was analyzed using SEM and AFM. Data were statistically analyzed using one-way ANOVA followed by Tukey’s HSD test. The results showed that gels containing papain and bromelain were more effective than the commercial gel in removing stains. SEM and AFM analysis indicated that papain was particularly effective for removing coffee stains, while bromelain was better for stains from natural juices. Healthy enamel has a Ra value of approximately 10 nm, which increases to about 40 nm after staining. Papain restored enamel roughness to approximately 8 nm for coffee stains and bromelain restored it to 11 nm for juice stains, delivering optimal results, while commercial gel ensures a roughness of about 15 nm after stain removal. CIELAB reveals notable changes in ΔE and WI after bleaching, revealing that papain gel is optimal for coffee stain removal and bromelain gel is optimal for natural juice stains. In conclusion, dental stains should be assessed by a dentist to determine the most suitable gel for achieving optimal results.

## 1. Introduction

The integrity of tooth enamel is influenced by the cyclic interactions with food and beverages, which exert both abrasive and erosive effects accompanied by microstructural deposits. Abrasion occurs when hard food particles rub against the enamel surface under masticatory forces [1,2]. The erosive effect has two primary components: direct erosion caused by acids in food and beverages—for example, citric acid [3,4,5,6], phosphoric acid [7,8], and—in some cases—carbonic acid [9,10]; and indirect acid demineralization, which arises from bacterial activity that decomposes food remnants adhered to the enamel surface [11,12,13], including colored stains [14,15].

Maintaining enamel integrity relies on consistent oral hygiene; specifically, tooth brushing with toothpaste after every meal. However, such stringent hygiene practices are often impractical in modern daily routines [16,17,18]. Daily activities often include the consumption of beverages such as coffee and natural or carbonated juices, without immediate cleaning afterwards. Both coffee and natural juice contain microstructural dispersoids with high adsorption tendencies to the enamel surface during drinking. The coalescence of these dispersoids forms adherent stains that are difficult to remove, even with evening tooth brushing [15,16,19,20,21].

The staining mechanism is well documented. Existing data indicate that proper oral hygiene reduces the occurrence of stains on the enamel surface, but cannot entirely prevent their proliferation unless strict protocols are followed. This challenge has driven the development of whitening gels, focusing on innovative formulations for effective stain removal. Many whitening gels rely on carbamide peroxide, which releases hydrogen peroxide upon contact with stains, enabling advanced stain removal. However, this process carries the risk of damaging the enamel surface due to intensive oxidation [22,23]. The effectiveness of these gels depends on precise dosing and the duration of application.

To address the shortcomings of peroxide-based gels, newer generations of enzymatic gels have been developed. These gels replace carbamide peroxide with specific enzymes capable of breaking down stain deposits. For instance, alkaline phosphatase can moderate the bleaching response of carbamide peroxide, reducing potential enamel damage [24,25,26]. Münchow et al. demonstrated the effectiveness of papain and bromelain in stain removal, showing their ability to clean while preserving enamel integrity, as corroborated by other studies [27,28].

Papain, a proteolytic enzyme derived from the latex of Carica papaya leaves and fruit, is a well-known natural remedy for treating infections, reducing inflammation, and alleviating pain. Its efficacy has been demonstrated in various conditions, including skin allergies, wound healing, the amelioration of digestive symptoms through its antibacterial effects, and the reduction of autoimmune skin eruptions [29,30].

Bromelain, an enzyme extracted from pineapple (*Ananas comosus*), belongs to the cysteine-protease group of hydrolytic enzymes. It can cleave peptide bonds within organic compounds due to its sulfhydryl functional groups [31]. Bromelain is wellknown for its use in treating burn injuries, providing antibacterial effects, and facilitating clean wound healing [32].

The functional groups in papain and bromelain enhance catalytic oxidation within bleaching gels, effectively targeting stain deposits. These enzymes disintegrate stain microstructures through their bioactive effects, improving removal efficiency.

The present study aims to evaluate the whitening efficiency of experimental gels containing papain and bromelain for the removal of coffee and natural juice stains, comparing their performance to a commercial carbamide peroxide-based bleaching gel. Microstructural and nanostructural changes will be analyzed using scanning electron microscopy (SEM) and atomic force microscopy (AFM). The morphological and topographic findings will be correlated with color measurements based on the CIELAB system. The study’s null hypothesis has two components: (1) experimental gels based on papain and bromelain are as effective as the commercial carbamide peroxide gel; and (2) there are no significant differences in the effects of papain and bromelain.

## 2. Results and Discussion

The incorporation of enzymatic bioactive compounds requires the formulation of a neutral gel to ensure the stability of the composition. The literature data suggest that quince juice can provide a stable matrix for this purpose [33,34]. Accordingly, the control gel (GC) features a matrix composed of PEG 400 mixed with freeze-dried whey, fluidized by the addition of Lubrizol and incorporating quince juice. The detailed preparation method for this control gel is described in one of our previous studies [35].

To evaluate the effects of the bioactive enzymatic compounds on stain removal, the enzymes were integrated into the control gel. The resulting formulations included G1, containing papain; and G2, containing bromelain. For comparative analysis, healthy enamel was used as the positive control, while enamel stained with coffee and natural juice served as the negative controls.

### 2.1. Micro- and Nanostructural Characterization of the Teeth Etalons

**Figure 1:**
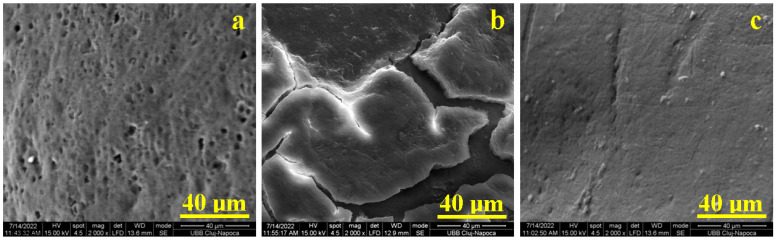
SEM images of teeth enamel etalon microstructure: (**a**) healthy–untreated, (**b**) coffee-stained, and (**c**) juice-stained.

The positive etalon exhibits a uniform microstructure, as shown in Figure 1a, with evidence of mild wear resulting from interactions with food during mastication. This observation aligns well with the previously reported literature [2]. Notably, some regions, particularly in the lower-middle section of Figure 1, display a honeycomb-like structure. These honeycomb formations consist of rounded pits with diameters ranging from 2 to 3 μm, indicative of the early stages of mild acid erosion. The literature reports that acid demineralized enamel typically exhibits a honeycomb structure, with unit cell diameters of approximately 5 μm [36,37]. Overall, the positive etalon demonstrates a well-preserved enamel surface, reflecting the benefits of good oral hygiene practices.

Coffee is a beverage containing fine particles of ground coffee beans dispersed in boiled water. These particles exhibit sticky behavior due to the composition of coffee, which includes approximately 30% polysaccharides, 13% proteins, and 0.8% fats and waxes; all activated by the hot water [38,39]. The fats and waxes serve as binding agents for the smaller polysaccharide particles, such as starch, leading to the formation of compact stain scales, as shown in Figure 1b. These scales have sizes ranging from 100–300 μm and form a complex structure interlocked with polyhedral cracks that propagate among them.

Natural juice, on the other hand, contains significant amounts of nano-sized fractions of plant-derived fibers such as cellulose, hemicellulose, and lignin from the fruit pulp [40]. These are combined with moderate amounts of polysaccharides and natural pigments [41] floating in a water base rich in sugars and natural acids; including citric acid, ascorbic acid, and beta carotene. The high sugar content facilitates the adhesion of dispersoids to the enamel surface, while the acids locally demineralize the outermost enamel layers. The SEM image in Figure 1c reveals that acid erosion accentuates pre-existing wear scratches. Additionally, all microstructural features of the enamel surface are coated by a uniform stain layer.

**Figure 2:**
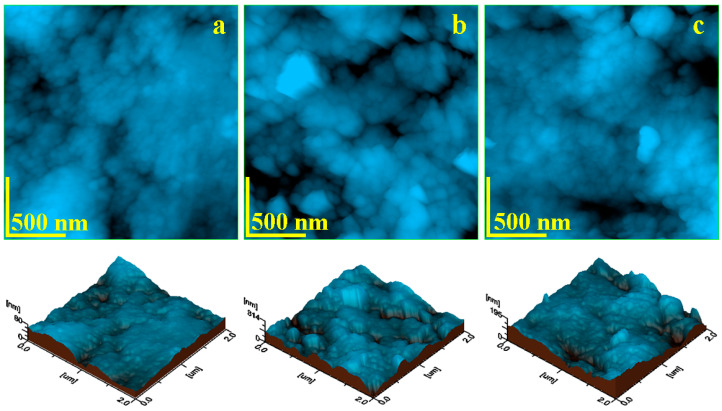
AFM topographic images of teeth enamel etalon nanostructure: (**a**) healthy–untreated, (**b**) coffee-stained, and (**c**) juice-stained. Three-dimensional profiles are presented below each topographic image.

The enamel nanostructure is located within the inner regions of each hydroxyapatite prism and consists of a densely packed arrangement of rounded nanostructural units with diameters of approximately 40 nm. These units are composed of tightly bonded hydroxyapatite crystallites, held together by a proteinaceous matrix [1]. As a result, the topography of healthy enamel exhibits a uniform and compact surface, with nanostructural units closely packed; as illustrated in Figure 2a. This structural organization contributes to a low surface roughness, characterized by values of approximately Ra = 9.42 nm and Rq = 11.7 nm.

The nanostructure of coffee stains exhibits a granular morphology, as shown in Figure 2b, consisting of small polysaccharides embedded within waxes. These components form submicron clusters with a boulder-like appearance, ranging in size from 200 to 400 nm. These adherent clusters completely obscure the natural enamel features, resulting in a significant increase in surface roughness, with Ra = 35.7 nm and Rq = 45.3 nm. The irregular arrangement of the coffee-stain nanoclusters creates localized gaps approximately 1 μm in length and 100 nm in width. Notably, there are no indications of acid demineralization associated with coffee stains.

In contrast, the nanostructure of natural juice stains reveals a more compact deposit that entirely covers the enamel’s natural features; as illustrated in Figure 2c. The nano-dispersoids, such as pigments and the finest pulp fractions, are embedded within a matrix of extremely fine fiber fragments. This compact structure obscures the visualization of individual fiber fragments, which is consistent with the literature findings on apple and carrot peels and pulp [40]. Figure 2c also highlights two significant demineralized depressions, located in the upper-right corner and the lower-middle area. These eroded regions exhibit irregular dendritic margins and range in size from 600 to 800 nm. While the dirt layer overlays these depressions, it does not fill them entirely. Consequently, the surface roughness increases further, with mean values of Ra = 44.9 nm and Rq = 58.9 nm.

The nanostructural findings indicate that coffee stains are less compact compared to natural juice deposits and do not cause direct acid demineralization. However, both types of stains significantly increase surface roughness, with natural juice stains resulting in higher roughness values due to localized demineralization.

### 2.2. Micro and Nanostructural Evidence of Stain Removal

**Figure 3:**
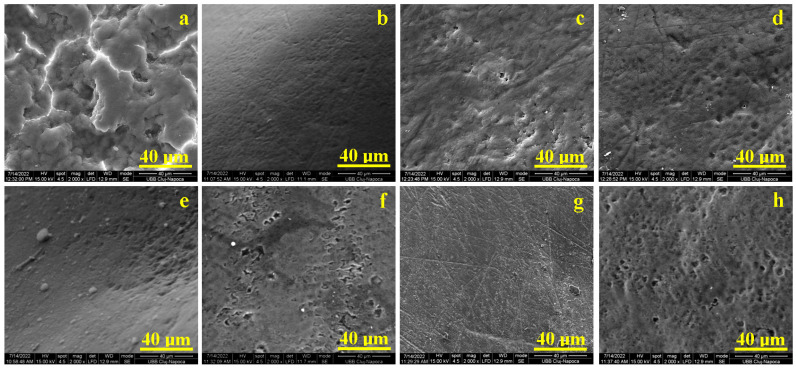
SEM images of the stained enamel microstructure. Coffee treated with: (**a**) GC; (**b**) G1; (**c**) G2; (**d**) GO. Juice treated with: (**e**) GC; (**f**) G1; (**g**) G2; (**h**) GO.

The GC moistens the coffee-stained surface, penetrating and swelling the scale structures while filling the interlocked cracks, as shown in Figure 3a. The Lubrizol component facilitates fluidization and gel penetration into the coffee scales. However, due to the absence of bioactive ingredients, the dirt deposits remain firmly attached to the enamel surface.

In the case of G1, which contains papain, the enzyme is effectively delivered through the gel into the fissures of the swollen coffee stains. It begins breaking down the scale structure. The SEM image in Figure 3b shows that the coffee stains have been completely removed, leaving a uniform, smooth, and compact enamel surface. The hydroxyapatite prisms appear well packed—similar to those in healthy enamel—with notable improvements, as no wear marks are observed. These results align with findings from a previous study [42].

G2, containing bromelain, also effectively penetrates the coffee stains through the Lubrizol-mediated action. The bioactive compound initiates the breakdown of the dirt microstructure. As shown in Figure 3c, bromelain successfully removes all visible coffee scales. However, residual wear marks are observed in a diagonal pattern from the upper-left to the lower-right corner of the image. These may be associated with secondary acid erosion marks caused by bacterial activity within the organic matter of the coffee scales. This observation warrants further advanced nanostructural investigation.

The Opalescence gel, based on carbamide peroxide, also infiltrates the fissures of the coffee scales and initiates disintegration through intensive oxidation. Following treatment according to the manufacturer’s instructions, the stains are completely removed, as shown in Figure 3d. However, the process slightly accentuates the wear marks on the enamel surface compared to its initial state.

The compact structure of the natural juice stain resists penetration attempts by the GC, as seen in Figure 3e. Without bioactive compounds, the gel fails to create the microstructural breaches necessary for stain removal.

When G1 comes into contact with the stain surface, papain takes immediate action, creating microstructural breaches that allow deeper penetration. This enzymatic action initiates the breakdown of the stain’s compact structure, leading to its complete removal, as shown in Figure 3f. However, wear marks on the enamel surface, partly accentuated by direct acid erosion from the natural juice, remain visible.

Bromelain in G2 also targets the natural juice stain upon contact, achieving advanced stain removal and notable recovery of the enamel surface; as observed in Figure 3g. The wear marks and acid erosion features are less pronounced following G2 treatment, suggesting localized remineralization. This effect may result from partial redistribution of hydroxyapatite within the enamel surface, as supported by prior studies [43,44].

The Opalescence gel effectively eliminates natural juice stains. However, the intense oxidative action of carbamide peroxide amplifies the acid demineralization structures caused by exposure to natural juice. Figure 3h shows that honeycomb-like structures cover most of the surface, with only a few incipient erosion spots that have not propagated in depth. This observation underscores the importance of evaluating the enamel’s condition before applying carbamide peroxide gel. Adjusting the exposure time to accommodate the enamel’s pre-existing condition is strongly recommended.

**Figure 4:**
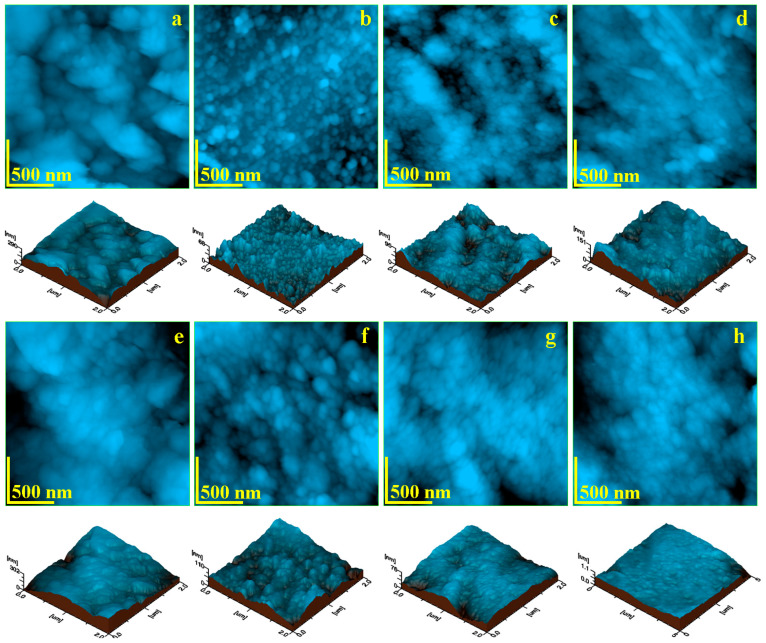
AFM topographic images of the stained enamel nanostructure: coffee treated with: (**a**) GC; (**b**) G1; (**c**) G2; (**d**) GO; and juice treated with: (**e**) GC; (**f**) G1; (**g**) G2; (**h**) GO.

The nanostructural analysis of the treated enamel surfaces was conducted using AFM. The resulting topographical images are presented in Figure 4.

The nanostructural topography of the coffee-stain scale, shown in Figure 4a, is affected by the penetration of the control gel into the intergranular fissures, resulting in observable swelling of the clusters. These clusters retain their boulder-like shapes, but their sizes increase to 300–600 nm. The swelling effect reduces the gaps in the fissures, which is reflected in a slight decrease in roughness (Rq ≈ 42.9 nm).

Papain penetration into the submicron clusters of dirt introduces amine and hydroxyl functional groups, which strongly react with the polysaccharides and waxes within the coffee scales, causing their nanostructural disintegration, as described in the literature by Münchow [27]. Consequently, the coffee dirt is completely removed, and the enamel nanostructure becomes clearly visible in Figure 4b. The nanostructural units are rounded, approximately 40 nm in size, and packed in a dense, uniform structure similar to healthy enamel. Papain exerts a gentle whitening effect on the enamel nanostructure, facilitating mild reorganization of the hydroxyapatite within the nanostructural units to repair any potential second-class acid demineralization marks. As a result, surface roughness decreases to Ra = 7.55 nm and Rq = 9.75 nm.

The sulfhydryl functional groups in bromelain are highly effective in disrupting the swollen clusters of coffee wax and embedded polysaccharides (e.g., traces of starch). Therefore, the coffee scales are completely removed at the nanostructural level; as seen in Figure 4c. The nanostructural units are rounded, with diameters around 40 nm, and are packed in a dense, compact structure typical of healthy enamel. Unfortunately, the coffee stains induce mild acid demineralization of the second type, which results in a wavy topography. This mild topographical unevenness does not affect enamel health but slightly influences the mean roughness values (Ra = 10.6 nm and Rq = 13.2 nm), which do not decrease as much as after G1 treatment.

Carbamide peroxide in the Opalescence gel has a strong stain-removal effect, completely destructuring the coffee scales through direct oxidation, as shown in Figure 4d. However, this intense action has unwanted consequences on the enamel surface topography, as it exacerbates the wear and acid erosion marks observed on healthy enamel in Figure 2a. Some oblique parallel scratches are evident on the upper and right sides of Figure 4d, and there are residual acid demineralization depressions on the left side of the image. This certainly affects the mean roughness values, which are Ra = 17.3 nm and Rq = 21.9 nm.

The control gel has a nanostructural moistening effect on the natural juice stain, causing the swelling of its components; as seen in Figure 4e. Short cellulose fibers, approximately 100–500 nm in length, are mixed with nanostructural pigments and fruit pulp remains. The swelling effect through humectation is supported by the literature data [40]. These nanostructural alterations cause a slow reduction in surface roughness due to the reduction of intergranular gaps from the swelling of nanostructural features (Ra ≈ 44.9 nm and Rq ≈ 55.0 nm). This is not enough to remove the stain, but certainly prepares the surface for the bioactive compounds.

The amine and hydroxyl active groups in papain attack the compact crust of the natural juice stain. The literature data show that amine groups functionalize the cellulose fibers, enhancing their microstructural features [45], while hydroxyl groups facilitate the dissociation of the nanostructural pigments and fruit pulp remains [46]. As a result, the cohesion of the nanostructural components of the stain is weakened, and the crust is penetrated. Figure 4f reveals the topography after bleaching with G1, showing a compact and dense structure of rounded nanostructural units that are well embedded within each other, with traces of acid demineralization induced by exposure to the natural juice. The mean roughness is Ra = 11.7 nm and Rq = 15.5 nm, close to the normal values for healthy enamel.

Lin et al. [47] showed that free sulfhydryl groups affect interaction forces with cellulose nanofibril surfaces and also influence thiol and amine bonds within pigments [48]. Bromelain in G2 thus enhances the superficial forces of the cellulose fibers while disrupting the nanostructural pigments, penetrating deep into the natural juice stain. This gentle removal process completely eliminates the stain, as shown in Figure 4g. The hydroxyapatite nanostructural units are rounded, approximately 40 nm in diameter, and packed in a dense, compact, and smooth structure. Surface roughness decreases to Ra = 9.95 nm and Rq = 12.1 nm, which is nearly identical to healthy enamel. This indicates that localized remineralization occurs due to self-healing mediated by the proteolytic enzyme in G2.

Figure 4h reveals the topography after Opalescence gel treatment, where the carbamide peroxide removes the natural juice stain through oxidative action. The enamel surface is free of juice stains but exhibits significant acid erosion marks. The natural juice exacerbates pre-existing acid erosion marks due to the natural wear of the tooth surface—such as the large depression on the left side of Figure 4h—and forms new eroded spots ranging from 100 to 200 nm in diameter, consistent with the literature [49]. Thus, the surface roughness is Ra = 15.2 nm and Rq = 21.5 nm.

Both micro- and nanostructural investigations converge and indicate that the experimental bioactive gels are more effective in stain removal than the commercial gel containing carbamide peroxide. Consequently, the obtained experimental results reject the first part of the null hypothesis. Furthermore, it was observed that the papain-based gel G1 is more effective for removing coffee stains, while the bromelain-based gel G2 is more effective for removing natural juice stains.

**Figure 5:**
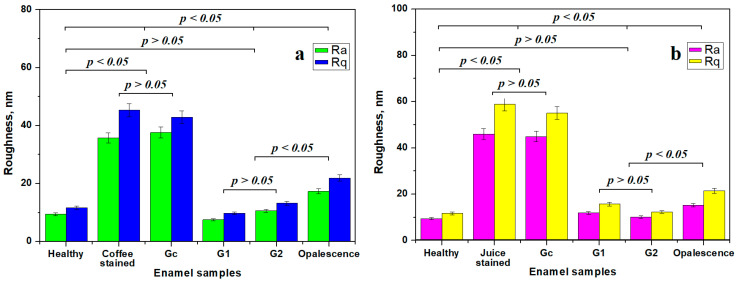
Mean roughness of the enamel surface depending on the applied treatment for the samples stained with: (**a**) coffee and (**b**) juice.

A quantitative measure of the qualitative observations, based on microscopic investigation, is provided by the surface roughness variation in Figure 5. Statistical analysis shows that the healthy enamel forms a statistically significant group with the samples treated with G1 and G2, proving the complete whitening success of these gels.

Stained samples (coffee and natural juice) form statistically relevant groups with the samples treated with the control gel, proving that it has no significant effect on stain removal. ANOVA followed by Tukey’s post hoc analysis reveals a significant statistical difference between the healthy enamel group and the stained enamel groups. Finally, the sample treated with Opalescence gel forms a distinct statistical group, which is different from the other mentioned groups, indicating that the stain removal was effective for both coffee and natural juice. However, the wear and acid demineralization features were enhanced.

### 2.3. Color Changes Assessment

**Figure 6:**
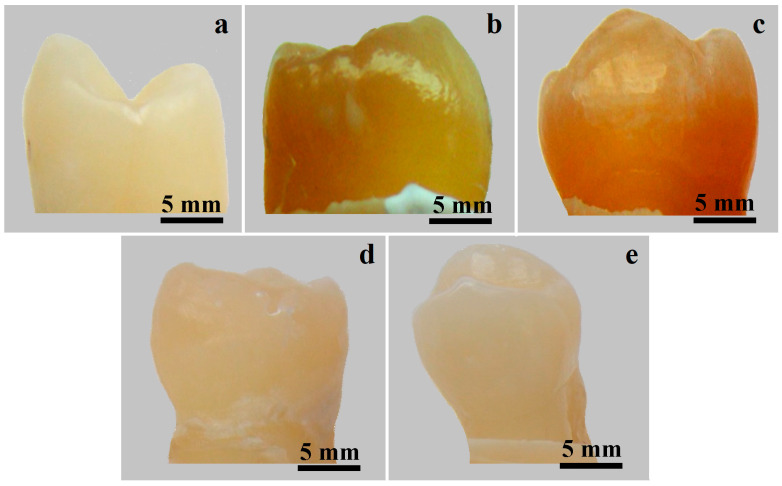
Photographs of the main teeth samples: (**a**) healthy–unstained, (**b**) coffee-stained, (**c**) natural-juice-stained, (**d**) coffee-stained bleached with G1 and (**e**) natural-juice-stained bleached with G2.

The results of the color differences before and after the bleaching process are presented in Figure 6. After the staining procedure, both in coffee and juice, the GC showed no statistically significant difference. However, minor differences in tooth color are attributed to the presence of quince juice. Quince contains natural acids (such as malic acid) that may help break down surface stains [50].

In the case of the experimental gels with proteolytic enzymes, they demonstrated a statistically significant whitening effect, indicated by a decrease in ΔE after the bleaching process (*p* < 0.05 in each case, for both intensive staining processes).

**Figure 7:**
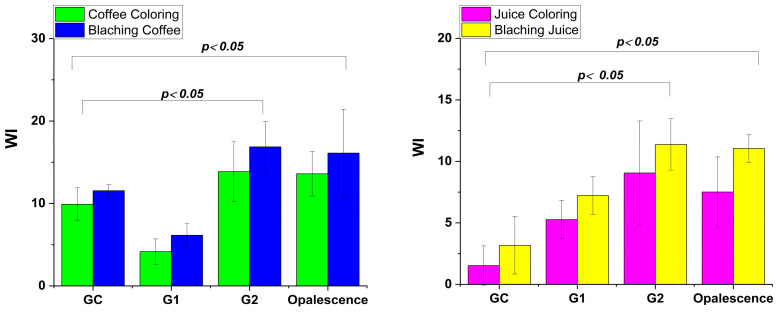
Mean color changes (ΔΕ) of the enamel surface depending on the applied treatment (coloring with coffee/juice and bleaching coffee/juice).

Papain gel G1, shows the highest ΔE* (Figure 7), making it most effective in removing discoloration caused coffee stains. Bromelain Gel G2 is effective for organic stains (fruit-based discoloration), but slightly less so than commercial gel.

Figure 6 highlights the differences between coffee and natural juice stains, which are readily distinguishable by professionals, enabling customized treatment strategies. Overall, our findings provide valuable insights for practitioners, allowing them to choose the most suitable bleaching gel for targeted stain removal, and ensuring optimal results. This approach supports the development of minimally invasive enamel whitening treatments tailored to each patient’s unique needs.

For the whitening index, there are significant differences among all four gels investigated, both in the case of the control gel between WI after bleaching and WI after staining with coffee and juice.

**Figure 8:**
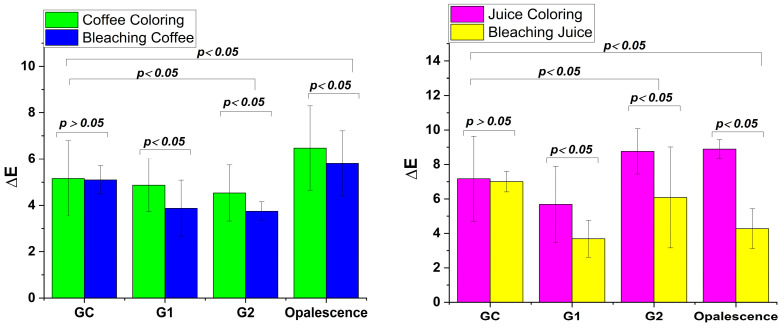
Mean whiteness index (WI) of the enamel surface depending on the applied treatment (coloring with coffee/juice and bleaching coffee/juice).

In the case of coffee staining, in Figure 8, the WI after bleaching shows similar values in G1 and Opalescence, as well as G2. For juice staining, the largest differences are observed for the commercial gel, Opalescence, followed by G1, G2, and GC.

*Limitations of the study*: The enamel surfaces of the teeth used in this study are influenced by various factors—including age, gender, hygiene practices, and dietary habits—which cannot be fully controlled. Additionally, the staining and whitening processes may not completely replicate real oral conditions. Additionally, a whitening treatment is also characterized by the reappearance of stains, the duration for which the whiteness of the teeth is maintained. This aspect could be mentioned in future studies, after a certain period of time after performing the whitening treatments. Nevertheless, this research provides valuable insights for developing new whitening gels based on proteolytic enzymes, which have the potential to deliver satisfactory results.

## 3. Conclusions

The bromelain and papain-based gels evaluated in this study proved effective in whitening stained tooth enamel. The Opalescence gel demonstrated comparable results to the experimental whitening gels in terms of color improvement but had a more significant impact on enamel structure.

Among the experimental gels, G1—which contained papain—was the most effective in removing coffee stains from enamel. On the other hand, G2—formulated with bromelain—excelled at eliminating stains from natural juices and mitigating acid erosion caused by citric acid, thereby enhancing the whitening index.

Incorporating whey into these gels provided a natural source of bioactive peptides with physiological and antimicrobial properties, further enriching the formulation. Structural analyses confirmed that all tested gels effectively removed deposits from tooth enamel, contributing to their overall whitening efficacy.

Enzyme-based gels have significant potential as natural treatments for safe and effective tooth whitening.

## 4. Materials and Methods

### 4.1. The Preparation of Whitening Gels Based on Proteolytic Enzymes

This study developed two whitening gels based on proteolytic enzymes. These enzymes were derived from natural extracts: papain was obtained from papaya (*Carica papaya*), and bromelain was extracted from pineapple (*Ananas comosus*).

**Figure 9:**
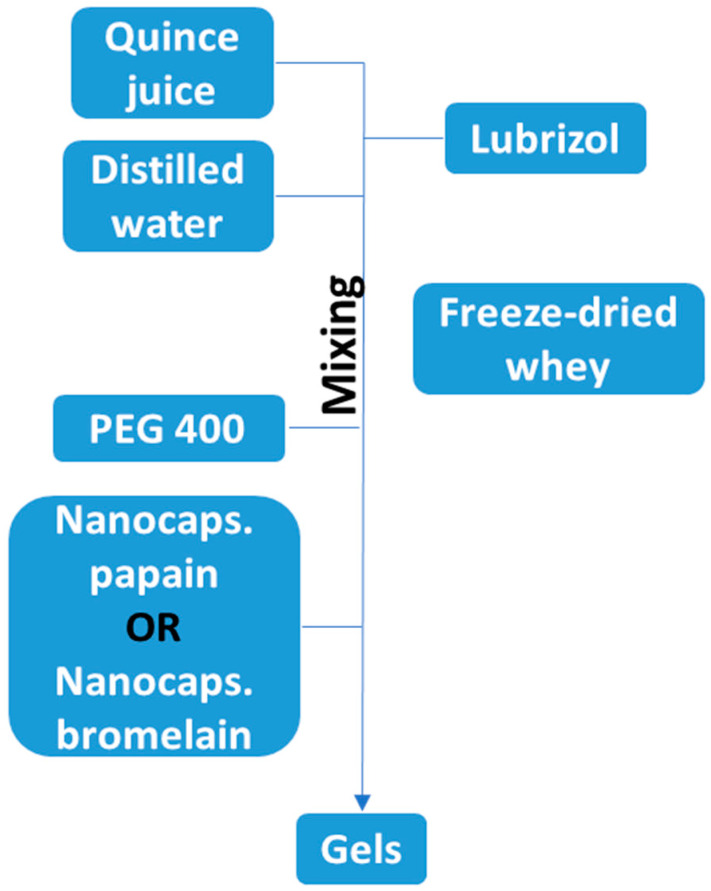
Flowchart of obtaining experimental gels.

The natural juices were extracted from the fruits (including peel and pulp) using a blender (Electrolux—model EBR 5050, Electrolux, Stockholm, Sweden) at 8000 rpm for 3 min. The resulting product was then filtered and centrifuged at 9000 rpm for 20 min. Activated charcoal was added to the resulting solution at a temperature of 40 °C to obtain a colorless solution. This solution was subsequently lyophilized using a Lyophilizer; Model Alpha 1-4LDPLUS, Martin Christ Gefriertrocknungsanlagen GmbH, Osterode am Harz, Germany (Figure 9).

Proteolytic enzymes (bromelain and papain) were encapsulated in biodegradable and biocompatible polymers using the interfacial deposition method with a polymer precursor, as described by Quintanar et al. [51].

To evaluate the whitening efficacy, the two experimental gels containing these enzymes were compared with a control gel without enzymes and a professional commercial tooth-whitening gel, Opalescence 15%.

### 4.2. Characterization of Whitening Gels

#### 4.2.1. Structural Analysis

The most commonly reported adverse effects following the tooth-whitening process include microstructural changes that can lead to increased surface roughness, reduced hardness, and even micro-cracks. This study analyzed the microstructure of dental surfaces and restorative composites before and after experimental whitening using two distinct techniques.

*Scanning electron microscopy*

The samples were examined using an Inspect-S scanning electron microscope (FEI Company, Hillsboro, OR, USA), before and after the bleaching protocol. The samples were placed in the microscope chamber under low vacuum, at room temperature (23 °C), and with an accelerating voltage of 30 kV. SEM images were recorded at a 2000× magnification level.

*Atomic force microscopy*

AFM imaging was performed with a Jeol Scanning Probe Microscope JSPM 4210 atomic force microscope, JEOL Ltd., Tokyo, Japan. The surfaces of the samples were investigated by scanning in Tapping Mode (intermittent tapping mode) using cantilevers of the NSC 15 type produced by MikroMasch, Innovative Solutions Bulgaria Ltd., Sofia, Bulgaria, with a resonance frequency of 330 kHz and a force constant of 48 N/m.

Each sample was analyzed in at least three distinct areas (over a scanning area of 2 μm × 2 μm) to ensure statistically valid characterization. Based on the topographic images and 3D profiles, surface roughness measurements were determined by calculating the $R_{a}\;$ and $R_{q}$ parameters. The $R_{a}$ and $R_{q}$ calculations, defined in Equations (1) and (2), involved the profile length (*l*) and the surface height (*z*) at point x.

(1) $$R_{a}=\frac{1}{l_{r}}\int_{0}^{l_{r}}\left|=\left(x\right)\right|dx$$

(2) $$R_{q}=\sqrt{\frac{1}{l_{r}}\int_{0}^{l_{r}}\left|z{\left(x\right)}^{2}\right|dx}$$

#### 4.2.2. Determining Whitening Gel Efficacy Through Color Measurement

Tooth color measurements were conducted digitally using the Vita Easy Shade Advance 4.0 spectrophotometer (Vita Zahnfabrik, Bad Sackingen, Germany). This device is a highly accurate tool for color determination in dentistry, designed to standardize and quantify tooth color based on the CIE ${Lab}^{*}$ color space, which measures lightness ($L^{*}$), red-green spectrum ($a^{*}$) and blue-yellow spectrum ($b^{*}$) [52]. Additionally, the new Whitening Index (WID) was calculated [53] using Formula (4).

(3) $$∆E*=\sqrt{{\left({∆L}^{*}\right)}^{2}+{\left({∆a}^{*}\right)}^{2}+{\left({∆b}^{*}\right)}^{2}}$$

(4) $${WI}_{D}=0.55L^{*}-2.32a^{*}-1.1b^{*}$$

Prior to each session, the spectrophotometer was calibrated according to the manufacturer’s instructions to ensure consistent and accurate readings. Each sample was positioned under controlled lighting conditions to minimize any external influences on color perception. The spectrophotometer tip was placed directly perpendicular to the surface, ensuring full contact to prevent light leakage. Three measurements were taken per site to calculate the average color values, ensuring the reliability of the readings. This method provided precise, reproducible color data, enabling an accurate assessment of color changes resulting from the whitening treatments applied in the study.

For this study, 80 extracted molars free from caries were used. To facilitate better handling of the enamel, the roots of the extracted teeth were fixed in cylinders with self-curing acrylic resin (Duracryl Plus, Spofadental Inc, Jičín, Czech Republic), with a diameter of approximately 15 mm, up to the enamel–dentin junction, so that the coronal surface remained exposed.

All enamel surfaces were randomly divided into two groups (n = 40) and subjected to a staining process by immersing the samples in either coffee or Tedi juice for 4 h/day, over 5 consecutive days. Afterward, the samples were washed and immersed in artificial saliva (Na_2_HPO_4_, NaHCO_3_, CaCl_2_, HCl-1M, H_2_O) at 37 °C.

Following the staining process, the samples were further divided into subgroups of n = 10 and treated with 4 different whitening gels (Table 1). On each tested surface, a thin and uniform layer of 0.5 mm whitening gel was applied and maintained for 4 h/day. After each application, the surface of the teeth was rinsed under a stream of water for 1 min and stored in plastic test tubes with artificial saliva that we maintained at a temperature of approximately 37 °C (specificity of the buccal cavity), using a laboratory water bath.

Data analysis: color changes were statistically analyzed to compare the effects of papain and bromelain treatments on enamel brightness and hue shifts, processed with OriginPro 2024b (OriginLab Corporation, Northampton, MA, USA) software, using one-way ANOVA followed by a Tukey’s HSD (Honestly Significant Difference) test. The null hypothesis states that all group means are equal.

## Figures and Tables

**Table 1:** Experimental whitening gels with proteolytic enzymes composition.

Gels	Lubrizol [%]	Freeze-Dried Whey [%]	PEG 400 [%]	Quince Juice [%]	Distilled Water [%]	Nanocaps. Papain [%]	Nanocaps. Bromelain [%]	pH
GC	6–8	1–3	15–17	48–50	28–30	-	-	7
G1	2–4	1–2	7–9	55–57	22–24	2–4	-	5
G2	6–8	1–3	-	48–50	30–32	-	5–7	5
GO	Glycerin: 20–30; Potassium nitrate: 3–5; Fluoride 0.11				40–50	Carbamide peroxide 15		6.5–7.5

Lubrizol (Lubrizol Advanced Materials, Inc., Brussels, Belgium), aerosil (SiO_2_—Sigma-Aldrich Chemie GmbH, Schnelldorf, Germany), polyethylene glycol (PEG 400—Sigma-Aldrich Chemie GmbH, Schnelldorf, Germany), GC—gel control, GO—Opalescence 15% (GC, Ultradent Products, Inc., South Jordan, UT, USA).

## Data Availability

The original contributions presented in the study are included in the article; further inquiries can be directed to the corresponding author.

## References

[1] Ablal M.A., Milosevic A., Preston A.J., Higham S.M. (2017). A novel approach to study in situ enamel erosion and abrasion lesions. J. Dent..

[2] Lei L., Zheng L., Xiao H., Zheng J., Zhou Z. (2020). Wear mechanism of human tooth enamel: The role of interfacial protein bonding between HA crystals. J. Mech. Behav. Biomed. Mater..

[3] Muntean A., Dârgău C.-M., Pacurar M., Neagoe S., Delean A.G. (2023). Effect of Remineralizing Agents on Shear Bond Strength of Orthodontic Brackets—In Vitro Study. Children.

[4] Dündar A., Şengün A., Başlak C., Kuş M. (2018). Effects of citric acid modified with fluoride, nano-hydroxyapatite and casein on eroded enamel. Arch. Oral Biol..

[5] Schulze K.A., Santucci N.M., Surti B., Habelitz S., Bhattacharyya M., Noble W. (2024). Evaluation of Enamel Volume Loss after Exposure to Energy Drinks. Oral.

[6] Abbassy M.A., Masoud A.I., Alsulaimani F.F., Bakry A.S. (2022). Effect of citric acid erosion on enamel and dentin and possible protection by a novel bioactive borate adhesive system. J. Dent..

[7] Jabbour Z., Kim M., Hayashi M., Kim R. (2023). Phosphoric Acid Etch Partially Restores the Initial Bond Strength of Composite to Silver Diamine Fluoride–Treated Enamel Using Universal Adhesives. Dent. J..

[8] Ghoubril V., Changotade S., Lutomski D., Ghoubril J., Chakar C., Abboud M., Hardan L., Kharouf N., Khoury E. (2022). Cytotoxicity of V-Prep Versus Phosphoric Acid Etchant on Oral Gingival Fibroblasts. J. Funct. Biomater..

[9] Chen J., Zhang Y., Yin I.X., Yu O.Y., Chan A.K.Y., Chu C.H. (2024). Preventing Dental Caries with Calcium-Based Materials: A Concise Review. Inorganics.

[10] Inchingolo A.M., Malcangi G., Ferrante L., Del Vecchio G., Viapiano F., Mancini A., Inchingolo F., Inchingolo A.D., Di Venere D., Dipalma G. (2023). Damage from Carbonated Soft Drinks on Enamel: A Systematic Review. Nutrients.

[11] Mosaico G., Pinna M., Grassi R., Orrù G., Scribante A., Maiorani C., Casu C., Nardi G.M., Butera A. (2024). Oral Health and Caries Prevention: How Tongue Hygiene Helps Maintain Balance of Microbiota and Overall Health in Pediatric Patients. Children.

[12] Wassel M.O., Khattab M.A. (2017). Antibacterial activity against Streptococcus mutans and inhibition of bacterial induced enamel demineralization of propolis, miswak, and chitosan nanoparticles based dental varnishes. J. Adv. Res..

[13] Liu D., Yolandani G.Y., Ma H., Ashokkumar M. (2023). Dynamic changes of microbial communities during natural solid-state fermentation of soybean meal and isolation of dominant bacteria for peptide production. Food Biosci..

[14] Manno S.H.C., Manno F.A.M., Ahmed I., Ahmed R., Shu L., Li L., Xu S., Xie F., Li W.L., Ho J. (2018). Spectroscopic examination of enamel staining by coffee indicates dentin erosion by sequestration of elements. Talanta.

[15] Kim S., Lee C.-H., Ma S., Park Y.-S. (2024). Whitening Efficacy of Toothpastes on Coffee-Stained Teeth: An Enamel Surface Analysis. Int. Dent. J..

[16] Han S., Kim S.J., Lee T., Jung H.-I., Lee K.E., Song J.S. (2024). Comparison of the Short Time Effect of an Oral Hygiene Education in Four Sessions via Quantitative Light-Induced Fluorescence Technology Versus Disclosing Agents in Children: A Randomized, Crossover Clinical Trial. Children.

[17] Alegría P.L., Landim S.F., Pérez Valdés V.A., Martínez Escudero N., Botelho J.N., Branco B.H.M., Villagrán F., Sandoval C., Marques D.C.d.S., Parrón Carreño T. (2024). Parental Stress in Autistic Children with Poor Oral Hygiene: A Pilot Study to Develop and Validate a Measurement Scale. Healthcare.

[18] Abe M., Mitani A., Hoshi K., Yanagimoto S. (2024). Screening for Systemic Diseases Associated with Dental Self-Care in Japanese Adolescents. J. Clin. Med..

[19] Bazzi J.Z., Bindo M.J.F., Rached R.N., Mazur R.F., Vieira S., Machado de Souza E. (2012). The effect of at-home bleaching and toothbrushing on removal of coffee and cigarette smoke stains and color stability of enamel. J. Am. Dent. Assoc..

[20] Fawad N.F. (2015). The effect of light activated bleaching versus orange juice on enamel’s micro-hardness. Tanta Dent. J..

[21] West N.X., Hughes J.A., Parker D.M., Newcombe R.G., Addy M. (1999). Development and evaluation of a low erosive blackcurrant juice drink 2. Comparison with a conventional blackcurrant juice drink and orange juice. J. Dent..

[22] Butera A., Maiorani C., Rederiene G., Checchi S., Nardi G.M. (2024). Evaluation of the Effectiveness of Different Types of Professional Tooth Whitening: A Systematic Review. Bioengineering.

[23] Shayegan A., Vozza I., Bossù M., Malikzade N. (2024). Impact of Exposure to Commonly Used Carbamide Peroxide on Dental Pulp Stem Cells. Appl. Sci..

[24] Ortiz M.I.G., Corrales Ureña Y.R., Aguiar F.H.B., Lima D.A.N.L., Rischka K. (2024). Enzymatically Driven Mineralization of a Calcium–Polyphosphate Bleaching Gel. Bioengineering.

[25] Satti M.K., Nayyer M., Alshamrani M., Kaleem M., Salawi A., Safhi A.Y., Alsalhi A., Sabei F.Y., Khan A.S., Muhammad N. (2023). Synthesis, Characterization, and Investigation of Novel Ionic Liquid-Based Tooth Bleaching Gels: A Step towards Safer and Cost-Effective Cosmetic Dentistry. Molecules.

[26] Puleio F., Fiorillo L., Gorassini F., Iandolo A., Meto A., D’Amico C., Cervino G., Pinizzotto M., Bruno G., Portelli M. (2022). Systematic Review on White Spot Lesions Treatments. Eur. J. Dent..

[27] Münchow E.A., Hamann H.J., Carvajal M.T., Pinal R., Bottino M.C. (2016). Stain removal effect of novel papain-and bromelain-containing gels applied to enamel. Clin. Oral Investig..

[28] Chhabile S., Vishwakarma P., Agrawal A., Pundkar S.R., Mali G., Patil S., Gupta S. (2024). Effectiveness of Papain-Based Organic Dentifrices Versus Commercial Whitening Dentifrice on Tea-Induced Tooth Stains: An In Vitro Study. Cureus.

[29] Kong Y.R., Jong Y.X., Balakrishnan M., Bok Z.K., Weng J.K.K., Tay K.C., Goh B.H., Ong Y.S., Chan K.G., Lee L.H. (2021). Beneficial Role of *Carica papaya* Extracts and Phytochemicals on Oxidative Stress and Related Diseases: A Mini Review. Biology.

[30] da Silva Melo A.E.C., de Sousa F.S.R., dos Santos-Silva A.M., do Nascimento E.G., Fernandes-Pedrosa M.F., de Medeiros C.A.C.X., da Silva-Junior A.A. (2023). Immobilization of Papain in Chitosan Membranes as a Potential Alternative for Skin Wounds. Pharmaceutics.

[31] Kansakar U., Trimarco V., Manzi M.V., Cervi E., Mone P., Santulli G. (2024). Exploring the Therapeutic Potential of Bromelain: Applications, Benefits, and Mechanisms. Nutrients.

[32] Pertea M., Poroch V., Ciobanu P., Filip A., Velenciuc N., Lunca S., Panuta A., Buna-Arvinte M., Luca S., Veliceasa B. (2021). Efficiency of Bromelain-Enriched Enzyme Mixture (NexoBrid™) in the Treatment of Burn Wounds. Appl. Sci..

[33] Najman K., Adrian S., Sadowska A., Świąder K., Hallmann E., Buczak K., Waszkiewicz-Robak B., Szterk A. (2023). Changes in Physicochemical and Bioactive Properties of Quince (*Cydonia oblonga* Mill.) and Its Products. Molecules.

[34] Müller-Heupt L.K., Wiesmann-Imilowski N., Kaya S., Schumann S., Steiger M., Bjelopavlovic M., Deschner J., Al-Nawas B., Lehmann K.M. (2023). Effectiveness and Safety of Over-the-Counter Tooth-Whitening Agents Compared to Hydrogen Peroxide In Vitro. Int. J. Mol. Sci..

[35] Mazilu A., Popescu V., Sarosi C., Dumitrescu R.S., Chisnoiu A.M., Moldovan M., Dumitrescu L.S., Prodan D., Carpa R., Gheorghe G.F. (2021). Preparation and In Vitro Characterization of Gels Based on Bromelain, Whey and Quince Extract. Gels.

[36] Poggio C., Ceci M., Beltrami R., Lombardini M., Colombo M. (2014). Atomic force microscopy study of enamel remineralization. Ann. Stomatol..

[37] Besnard C., Marie A., Sasidharan S., Harper R.A., Marathe S., Moffat J., Shelton R.M., Landini G., Korsunsky A.M. (2023). Time-Lapse In Situ 3D Imaging Analysis of Human Enamel Demineralisation Using X-ray Synchrotron Tomography. Dent. J..

[38] Buelna-Chontal M. (2024). Coffee: Fuel for Your Day or Foe for Your Arteries. Antioxidants.

[39] Pyrzynska K. (2024). Useful Extracts from Coffee By-Products: A Brief Review. Separations.

[40] Filip M., Vlassa M., Petean I., Țăranu I., Marin D., Perhaiță I., Prodan D., Borodi G., Dragomir C. (2024). Structural Characterization and Bioactive Compound Evaluation of Fruit and Vegetable Waste for Potential Animal Feed Applications. Agriculture.

[41] Szot I., Łysiak G.P., Sosnowska B. (2024). The Beneficial Effects of Anthocyanins from Cornelian Cherry (*Cornus mas* L.) Fruits Their Possible Uses: A Review. Agriculture.

[42] Moldovan A., Cuc S., Gasparik C., Sarosi C., Moldovan M., Ilie N., Petean I., Rusu L.M., Ionescu A., Pastrav M. Effect of Experimental Bleaching Gels With Enzymes on Composite and Enamel. Int. Dent. J..

[43] Nath S.J.C., Fu Y., Li K.C., Loho T., Loch C., Ekambaram M. (2024). A Comparison of the Enamel Remineralisation Potential of Self-Assembling Peptides. Int. Dent. J..

[44] Liao J., Qiu J., Lin Y., Li Z. (2024). The application of hydrogels for enamel remineralization. Heliyon.

[45] Doostali M., Gholami Z., Sanaei D., Kazembeigi F., Ghasemi M., Ahmadi S., Javid A., Sarafraz M., Adiban M. (2023). Amino-functionalized cellulose fibers recovered from newspaper waste for efficient adsorption of crystal violet: Optimization using central composite design. Mater. Today Commun..

[46] Groeneveld I., Kanelli M., Ariese F., Bommel M.R. (2023). Parameters that affect the photodegradation of dyes and pigments in solution and on substrate—An overview. Dye. Pigment..

[47] Lin J., Yang J., Qi X., Shen M., Xie J. (2024). Effect of cellulose nanofibrils on formation, interactions and gelation properties of chickpea protein isolate emulsion gels. Food Hydrocoll..

[48] Passamonti S., Sottocasa G.L. (1990). The sulfhydryl groups responsible for bilitranslocase transport activity respond to the interaction of the carrier with bilirubin and functional analogues. Biochim. Biophys. Acta (BBA)-Biomembr..

[49] Chabuk M.M.G., Al-Shamma A.M.W. (2023). Surface roughness and microhardness of enamel white spot lesions treated with different treatment methods. Heliyon.

[50] Pei R., Xiao C., Zhu Y., Yao J., Cheng Y. (2022). Evaluation of tea stain removal efficacy of ficin. Food Sci. Technol..

[51] Quintanar-Guerrero D., Allémann E., Fessi H., Doelker E. (1998). Preparation techniques and mechanisms of formation of biodegradable nanoparticles from preformed polymers. Drug Dev. Ind. Pharm..

[52] Griber Y.A., Samoilova T., Al-Rasheed A.S., Bogushevskaya V., Cordero-Jahr E., Delov A., Gouaich Y., Manteith J., Mefoh P., Odettin V.J. (2024). “Playing” with Color: How Similar Is the “Geometry” of Color Harmony in the CIELAB Color Space across Countries?. Arts.

[53] da Rocha B.G.P.M., Ruiz-López J., Pérez M.M., Gaidarji B., Frasson G.T., Durand L.B. Effectiveness and one-year whiteness stability of different in-office bleaching agents and alternative protocols. J. Prosthet. Dent..

